# Aortic Valve Replacement in Adult Patients with Decellularized Homografts: A Single-Center Experience

**DOI:** 10.3390/jcm12216713

**Published:** 2023-10-24

**Authors:** Alexandra Andreeva, Iuliana Coti, Paul Werner, Sabine Scherzer, Alfred Kocher, Günther Laufer, Martin Andreas

**Affiliations:** Department of Cardiac Surgery, Medical University of Vienna, Währinger Gürtel 18-20, 1090 Vienna, Austria; iuliana.coti@meduniwien.ac.at (I.C.); paul.werner@meduniwien.ac.at (P.W.); sabine.scherzer@meduniwien.ac.at (S.S.); alfred.kocher@meduniwien.ac.at (A.K.); guenther.laufer@meduniwien.ac.at (G.L.); martin.andreas@meduniwien.ac.at (M.A.)

**Keywords:** aortic valve disease, surgical aortic valve replacement, homografts, decellularization

## Abstract

Background: decellularized aortic homografts (DAH) represent a promising alternative for aortic valve replacement in young adults due to their low immunogenicity and thrombogenicity. Herein, we report our midterm, single-center experience in adult patients with non-frozen DAH from corlife. Methods: safety, durability, and hemodynamic performance were evaluated according to current guidelines in all consecutive patients who had received a DAH at our center since 03/2016. Results: seventy-three (mean age 47 ± 11 years, 68.4% (*n* = 50) male) patients were enrolled. The mean diameter of the implanted DAH was 24 ± 2 mm. Mean follow-up was 36 ± 27 months, with a maximum follow-up of 85 months and cumulative follow-up of 215 years. No cases of stenosis were observed, in four (5.5%) cases moderate aortic regurgitation occurred, but no reintervention was required. No cases of early mortality, non-structural dysfunction, reoperation, valve endocarditis, or thrombosis were observed. Freedom from bleeding and thromboembolic events was 100%; freedom from re-intervention was 100%; survival was 98.6% (*n* = 72). Conclusions: early and mid-term results showed low mortality and 100% freedom from reoperation, thromboembolic events, and bleeding at our center. However, in order for this novel approach to be established as a valid alternative to aortic valve replacement in young patients, long-term data are required.

## 1. Introduction

Valvular heart disease (VHD) is a serious public health issue with an estimated prevalence of 2.5% [1,2]. Aortic valve disease, including aortic stenosis and regurgitation, comprises a large number of VHD cases [3]. Prevalence of aortic stenosis varies depending on the region and is drastically increasing with age, from 0.2% in patients in their 50s to 9.8% in 80–89 years group [4]. Bicuspid aortic valve, the most common type of congenital heart disease with 0.5–0.8% prevalence, is the main reason for surgery in younger patients with a mean age of 50 years at the time of the procedure [5,6,7]. Significant aortic valve regurgitation can be found in 0.5% of the population, with its incidence increasing with age [8].

Valve replacement is the only treatment option in severe cases of aortic valve stenosis and can be performed using different types of prostheses, but the choice of conduit in young adults still remains controversial. The attractiveness of a mechanical prostheses owing to their durability is limited by the thrombotic risk and the necessity of lifelong anticoagulation, which is associated with the increased risk of bleeding and is not acceptable for some patient groups (i.e., women planning to have children) [9,10]. On the contrary, biological prostheses do not require anticoagulation, but have a limited durability, which may require reintervention, especially in younger patients [11,12,13].

Decellularized homografts (DH) have been used since the early 2000s and have already shown good outcomes in younger patients with respect to survival and hemodynamic performance [14,15,16,17]. Compared to conventional homografts, different schemes of enzymatic and detergent removal of the donor cells are applied to reduce the immunogenicity of the homograft, potentially increasing the durability of the valve [18]. Importantly, grafts used herein are never frozen, which might protect structural integrity. Furthermore, spontaneous recellularization of DH was observed [19]. Low immunogenicity might not only contribute to the extended durability of decellularized homografts but is beneficial for younger patients who may require an organ transplantation in the future [20]. Therefore, not only freedom from lifelong anticoagulation, low immunogenicity, and good hemodynamic characteristics, but also a possible regeneration of the valve on the homograft matrix are potential major advantages of DH. Early results from multicenter prospective European trial ARISE demonstrated safety and efficacy of decellularized homografts, reporting low rates of adverse events and comparable outcomes to the Ross procedure after 2.5 years of follow-up [21]. As one of the main contributing centers, we herein report our single center experience with decellularized aortic homografts since 2016.

## 2. Materials and Methods

### 2.1. Patients and Data Collection

All adult patients undergoing aortic valve replacement with decellularized aortic homografts (DAH) between 2016 and 2023 at our center were included into this retrospective single center cohort study. Patients below the age of 65 who were not in favor of a mechanical aortic prosthesis were offered a decellularized aortic homograft as an alternative. Furthermore, patients below 40 years of age had the additional option of a Ross procedure. Patients were examined preoperatively, at discharge, and followed up with yearly after the procedure. The follow-up echocardiography exams were performed by institutional cardiologists.

Data were collected retrospectively through an institutional database. Adverse events and repeat procedures were acquired during follow up visits in an outpatient clinic and telephone calls. Mortality data were collected from the Austrian Federal State Agency and from the clinic’s database. The current study was approved by institutional review (Ethics Committee approval N°2201/2016 from 10 January 2017). Study enrollment was concluded on 30 November 2022

### 2.2. Endpoints

The primary endpoint of the study was determined to be all-cause mortality. Secondary endpoints, including reintervention, reoperation, structural valve deterioration (SVD), non-structural valve deterioration (non-SVD), bleeding, embolization, and thrombosis were reported according to current guidelines for adverse events reporting after valve interventions [22]. Additionally, the time between intervention and onset of adverse events was analyzed.

### 2.3. Operative Technique

**Figure 1:**
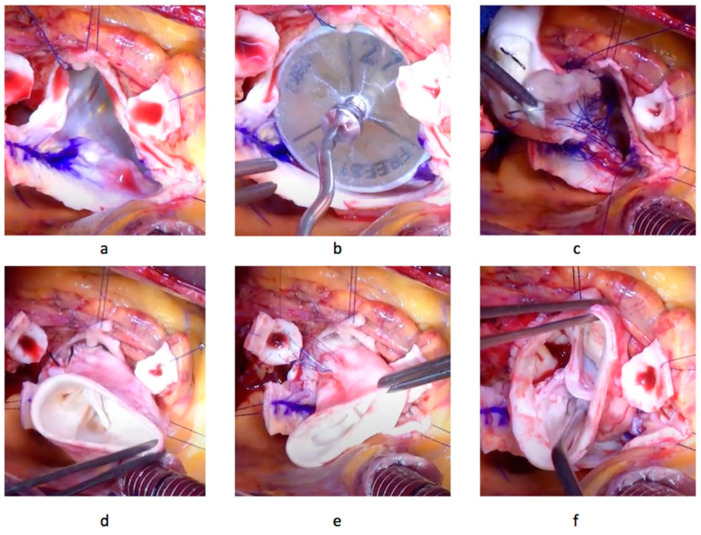
Surgical steps during aortic homograft implantation. (**a**) The aortic root is prepared for implantation of the DAH: the old valve and the coronary buttons are cut out and the marks corresponding to the commissures are set; (**b**) positioning of the sizer, the commissure marks set on the root match with those on the sizer; (**c**) proximal anastomosis of the DAH using three prolene sutures; (**d**) finished proximal anastomosis; (**e**) implantation of the left coronary artery into the homograft wall in the anatomically correct position; (**f**) inspection of the homograft. The left coronary artery is implanted, the implantation of the right coronary artery and the distal anastomosis will follow.

All patients included herein underwent total aortic root replacement with decellularized aortic homografts according to previously described technique [23]. After a median sternotomy and establishment of cardiopulmonary bypass and retrograde cardioplegia application, the aorta is cross-clamped and transected. After inspection of the aortic valve, its leaflets are excised and the aortic root is prepared (Figure 1).

Coronary ostia buttons are marked and prepared. The decellularized aortic homograft is inspected and implanted as a total root. The proximal anastomosis is performed with three 4-0 polypropylene sutures. Coronary ostia are anastomosed in standard fashion using a 6-0 polypropylene suture in continuous technique. The distal anastomosis between homograft and ascending aorta is performed using continuous 5-0 polypropylene sutures. After de-airing, the cross-clamp is opened, and the aortic valve is assessed via transesophageal echocardiography. All aortic homografts were decellularized according to a previously described protocol [24].

### 2.4. Data Analysis

Descriptive analysis of the study cohort was performed. Mean values and standard deviation were used to characterize continuous variables with a normal distribution. Continuous variables with a non-normal distribution were characterized by median and interquartile range. The Kaplan–Meier survival estimate (confidence interval 95%) was used to analyze mortality and risk of adverse events in patients receiving decellularized aortic homografts. Statistical analysis was carried out in IBM SPSS Statistics 29.0.0.

## 3. Results

Between 2016 and 2023, 73 adult patients received a decellularized aortic homografts at our center; 68% (*n* = 50) were male (Table 1).

Mean age at procedure was 47.0 ± 11.4 years. A total of 64.4% (*n* = 74) of the patients had a bicuspid aortic valve (BAV), with 9 (12%) patients having previously undergone an intervention or operation on the aortic valve: 5 (7%) patients had a valvulotomy, 2 (3%) patients had an aortic valve replacement with mechanical and biological prosthesis, 1 (1%) patient underwent a Ross procedure, and 1 (1%) patient had a balloon valvuloplasty. Two (3%) patients had more than one intervention or operation: one patient, after undergoing the Ross procedure, underwent a Yacoub procedure, and one patient, after undergoing a balloon valvuloplasty, had a further valvulotomy. Median EuroScore II was 1.8% [0.8, 2.9]. In 27 (37%) cases, the main indication that the aortic valve required replacement was severe aortic stenosis, in 24 (33%) cases it was severe aortic regurgitation, 21 (29%) patients had mixed aortic valve disease, and 1 patient received a decellularized aortic homograft due to an ascending aortic aneurysm and intolerance to the previously implanted mechanical prosthesis.

The mean duration of cardiopulmonary bypass (CPB) and aortic cross-clamp (ACC) was 163 ± 57 min and 122 ± 37 min, respectively. The mean diameter of the implanted DAH was 24 ± 2 mm. Concomitant procedures were performed in 39 (53%) cases. Reduction plasty of the ascending aorta was performed most commonly (*n* = 23, 32%), followed by coronary artery bypass grafting (CABG) in 8% of the patient cohort (*n* = 6), and additional ascending aorta replacement in 4 (6%) cases. Other concomitant procedures included atrial septum defect closure and mitral and tricuspid valve repair. Intraoperatively, in 5 (7%) cases, bleeding required a 2nd clamping of the aorta and was resolved by additional suturing with a pericardial patch: in two cases, bleeding occurred in the left ostium, in one case at the reduction plasty suture of the aorta with a small tear of the homograft, in one case at the proximal anastomosis, and in one case at distal anastomosis. In one (1%) case, coronary kinking after reimplantation of the left main artery led to subsequent coronary ischemia and, ultimately, bypass surgery. All of the patients could be weaned from CPB successfully, and none of them required an ECMO implantation perioperatively. One patient developed a complete atrioventricular block postoperatively and, subsequently, underwent a permanent pacemaker implantation on the 8th postoperative day. One patient developed postoperatively torsade de pointes ventricular tachycardia and ROCS after CPR on the 2nd post-op day; his further postoperative course was uncomplicated. One patient had a subxyphoidal drainage of pericardial effusion on the 8th postoperative day.

**Figure 2:**
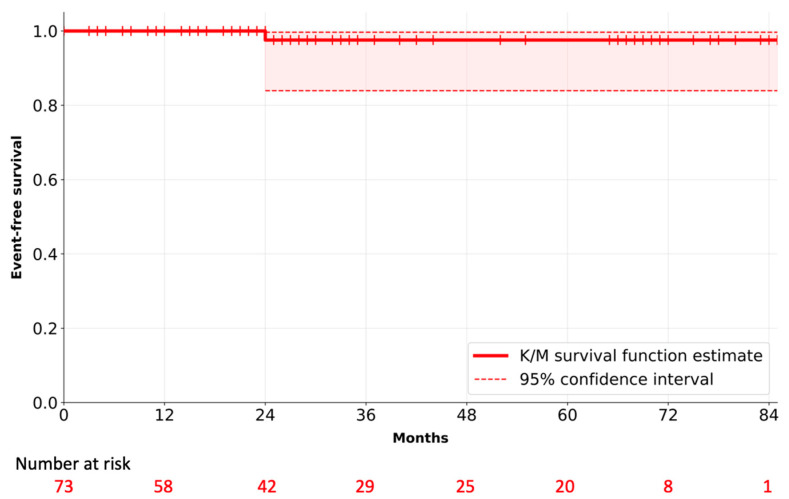
Kaplan–Meier event-free survival curve for decellularized aortic homografts.

Mean length of time between intervention and follow-up for survival of the study cohort was 36 ± 27 months, with a maximum follow-up interval of 85 months and cumulative follow-up of 215 years. Mean follow-up for adverse events was 27 ± 27 months. There were no cases of in-hospital or early mortality, overall survival was 98.6% (*n* = 72). (Figure 2).

One (1%) patient with terminal kidney disease died during Hemodialysis Reliable Outflow (HeRO) graft implantation after 10 years on dialysis and multiple shunt thrombosis and infections. There were no cases of bleeding, valve thrombosis, or embolization observed. None of the patients developed endocarditis postoperatively. The mean gradient at the last follow-up was 5.9 ± 3.6 mmHg, range 2–20 mmHg. Mean gradients over time were stable with no cases of stenosis of decellularized aortic homografts. Mean gradients (SD) at 1, 2, 3, 4, 5, 6, and 7 years were 6.2 (5.5), 6.6 (4.0), 5.7 (3.0), 6.7 (4.5), 6.1 (3.1), 10 (1.9), and 6.3 (2.5) mmHg, respectively. In 4 (6%) cases, moderate aortic regurgitation (SVD Stage 2r after D. Dvir et al. [25]) was observed during follow-up, the insufficiency of the valve remained constant during further observation, and patients were asymptomatic, not requiring any reintervention. Freedom from any type of reoperation and reintervention was 100%. No cases of non-structural dysfunction, structural degeneration, valve endocarditis, thrombosis, or re-intervention were observed.

## 4. Discussion

Herein, we evaluated mid-term outcomes for aortic decellularized homografts implanted at our center between 2016 and 2023.

No early or perioperative mortality was observed, with overall survival after a mean follow-up of 36 months being 98.6% (*n* = 72). One multimorbid (end-stage kidney disease with more than 10 years on hemodialysis, adipositas per magna) patient died after multiple infections and thrombosis of permanent dialysis catheter during implantation of a new hemodialysis reliable outflow graft. We observed no structural valve deterioration leading to reintervention or explantation of the valve (freedom from significant SVD = 100%).

These results are comparable with the mortality reported in the literature for aortic valve replacement, whereas the choice of the optimal prosthesis for AVR remains a challenge with each of the options bearing its disadvantages [26,27,28,29]. Five-year results of prospective randomized Veterans Affairs Trial reported 72 ± 3% survival for aortic valve replacement using mechanical prostheses and 70 ± 4% for bioprostheses in 1987; since then, the standard of treatment and postoperative care has significantly improved [30,31]. Therefore, the reported survival after AVR in adults under 50 years of age with mechanical or bioprostheses in a big retrospective analysis of more than 5000 patients was 94.0% (95% CI, 92.4–95.2%) and 92.6% (95% CI, 90.9–94.0%), respectively, five years after the medical procedure [32]. A meta-analysis of bioprotheses in 2686 nonelderly adults demonstrated a 91.6% survival within the first 5 years following the procedure, dropping to 58.7% 20 years after the procedure. Freedom from SVD or intervention was 98.8% and 96.8%, respectively, five years following the procedure and 30% and 29%, respectively, 20 years following the procedure, suggesting the need for the majority of patients to receive at least one repeat procedure in their lifetime, and durability being the main limitation of bioprostheses [33]. Noticeably, the rate of reinterventions and SVD varies depending on the bioprostheses type [34]. In our study cohort, no cases of SVD leading to reinterventions were observed.

A meta-analysis of a mechanical AVR in 5728 non-elderly patients demonstrated, once again, a high risk of thromboembolic and bleeding events (0.9% and 0.85%, respectively, per year) and suboptimal survival with late mortality risk of 1.55%/year [35]. Freedom from thromboembolism and bleeding in our study was 100%.

The Ross procedure represents another option for AVR in young adults. We previously showed excellent results with the Ross procedure, also compared to mechanical valves [36]. We reported a 96%, 94%, and 91% survival 5, 10, and 15 years, respectively, following the procedure, which in adults was not significantly different from the survival of the general population. A meta-analysis of 6892 adults undergoing the Ross procedure reported similar outcome with 91.9% survival, 83.9% freedom from autograft reintervention, and 91.0% freedom from RVOT reintervention 15 years following the procedure [37]. A total of 20 years following surgery, the Ross procedure offers a good survival of 87.3%. Additionally, 21.5% incidence of autograft reintervention may be reduced by autograft wrapping techniques [38]. Although the Ross procedure has a comparable mortality and is also widely used in our department despite its technical complexity, DAH could be a good alternative.

Our results are comparable with the ones reported in the literature with these specific grafts and indicate that DAH are a safe and reproducible alternative for AVR in young adults. Da Costa et al. reported a series of 41 patients receiving DAH with a median age of 34 years with relatively high (7%) early mortality and 90% survival after a mean follow-up of 19 months. No cases of severe SVD or reinterventions on DAH were observed; importantly, the decellularized grafts showed no calcification in follow-ups [39]. Another group reported no early mortality, low postoperative gradients, and low panel reactive antibody response at mean follow-ups of 30 months; however, these grafts were additionally cryopreserved [40].

Early results from the prospective ARISE trial and registry (*n* = 223), the biggest cohorts of DAH to date, report similar outcomes to those presented in the current study after a mean follow-up of 1.54 ± 0.81 years for prospective arm and 2.60 ± 2.13 years for the ARISE registry cohort [21]. A total of 36% of the patients included in current analysis were reported within early results of the prospective trial. A total of 38% of registry cohort were pediatric patients, accordingly the mean age of the cohort was lower than the one of this study: 28.7 ± 19.8 years in ARISE vs. 47.0 ± 11.4 years in our cohort. The authors reported very low rates of adverse events, comparable to results reported herein. Reported freedom from death, endocarditis, reoperations, bleeding, and thromboembolic events 5 years following the procedure were 98.2 ± 0.9%, 97.3 ± 2.2%, 90.8 ± 4.0%, 99.5 ± 0.5%, and 99.5 ± 0.5 respectively. Similarly, we report 98.6% survival and 100% freedom from endocarditis, reoperations, bleeding, and thromboembolic events. The ARISE registry cohort, however, included 47% of previously operated patients, while, in our study cohort, only 12% underwent an intervention or operation on the aortic valve previously.

A total of 32% (*n* = 23) of patients underwent a reduction plasty of the aorta, while 16 of them (69.6%) had a bicuspid aortic valve. These results are not surprising considering the fact that BAV is recognized not only as a valve pathology, but rather as a valvulo-aortopathy [41]. Twenty percent of young patients with BAV were shown to reach >40 mm diameter of ascending aorta at 9-year follow-ups following BAV diagnosis [42]. Another study demonstrated that 30% of asymptomatic young patients with BAV and mild valvular disease developed a dilation of ascending aorta of more than 40 mm 10 years after the procedure [43]. Aortic dilation in BAV patients has a higher risk of aortic aneurysm (≥45 mm diameter of tubular ascending aorta or root), which is 80 times higher than the risk of developing an aortic aneurysm in the general population. Furthermore, BAV patients are subject to a higher risk of aortic dissection and elective aortic repair [44]. BAV patients with moderate or greater aortic dilation who underwent isolated AVR often experience later dissection [45]; therefore, according to current guidelines [46], aortic repair should be performed concomitantly to AVR if the aortic diameter is >45 mm. Potentially, not only the reduction plasty of the aorta that was commonly performed in our study population, but the use of decellularized homografts itself as a root replacement technique might be beneficial and prevent future dilation and/or dissections in BAV patients.

Importantly, an improved durability of decellularized homografts compared to cryopreserved non-decellularized homografts is expected. Durability of cryopreserved grafts is limited, especially in younger patients [46]. Significantly higher freedom from explantation and a lower structural valve degeneration compared to cryopreserved pulmonary homografts 10 years following intervention was reported [47]. A study published in 2016 by Helder et al. [48] reported freedom from reoperation 10 years on for 51% (95% CI, 34–76%) of 41 patients implanted between 2002 and 2004. However, the decellularization technique of investigated grafts is different from the one used in our study, and the number of patients was limited. Nevertheless, longer follow-ups for these specific grafts is of outmost importance.

An intrinsic benefit of the decellularized homografts Is their low immunogenicity [18,19,20]. This is of utmost importance for patients who may later require organ transplantation [49]. Some patients, especially pediatric ones, with signs of early degeneration of decellularized homografts, seem to experience an immune response despite efficient donor cell removal [50,51]. Nonetheless, decellularized homografts in pediatric and very young adults demonstrate a significantly higher freedom from SVD and endocarditis 10 years following intervention compared to bovine jugular vein conduits [52].

The main limitation of this study is the lack of long-term outcomes, which is invaluable for evaluation of the durability and performance of decellularized aortic homografts. Our center will continue to follow up with patients enrolled in this study to further evaluate the hemodynamic characteristics of DAH. Furthermore, the access to this type of bioprostheses is limited by the low number of grafts available for decellularization and this type of AVR can be offered in only few centers worldwide.

Cost-effectiveness of decellularized homografts deserves consideration, considering that the price of a decellularized aortic homograft is approximately EUR 20,000. However, freedom from reoperation with valve explantation, which has been observed so far, reduced the rate of adverse events, and the expected longer durability compared to conventional grafts are of importance; decellularized homografts, therefore, might be more cost-effective in the long-run compared to other available valve substitutes, as the meta-analysis by Huygens et al. suggests [53].

Due to limited durability of bioprostheses, repeat procedures (re-SAVR or valve-in-valve TAVR and TAVR-in-TAVR) are inevitable for young adults throughout their life, so that the initially attractive cost-effectiveness of bio-SAVR may be diminished by the costs incurred for readmission, reintervention, and new prostheses needed by the patient. It has been demonstrated that even in elderly patients, decellularized homografts are more cost-effective than SAVR and TAVR due to reduced adverse events rates [54]. Also, being sold at a higher price than bioprostheses, decellularized homografts remain not only cost-effective, but, most importantly, demonstrate a quality-of-life gain for the patients.

## 5. Conclusions

Early and mid-term results showed low mortality and 100% freedom from reoperation, thromboembolic events, and bleeding at our center. Future studies are needed to evaluate the long-term performance of the DAH, which could be a safe and efficient alternative to the routine techniques of aortic valve replacement in young adults.

## Figures and Tables

**Table 1:** Baseline characteristic of the study cohort. DAH—decellularized aortic homografts, SD—standard deviation, IQR—interquartile range.

Baseline Characteristics	DAH
Patients, *n*	73
Age at surgery, y, mean (SD)	47.0 (11.3)
Sex, male, %	68.4
Weight, kg, mean (SD)	81.1 (20.1)
Height, cm, mean (SD)	173.6 (10.4)
Body mass index, kg/m^2^, mean (SD)	26.8 (5.7)
EuroScore II, %, median (IQR)	1.8 [0.8, 2.9]
Hypertension, *n* (%)	24 (32.9)
Chronical lung disease, *n* (%)	2 (2.7)
Renal insufficiency, *n* (%)	1 (1.4)
Hemodialysis, *n* (%)	1 (1.4)
Diabetes, *n* (%)	3 (4.1)
Dyslipidemia, *n* (%)	19 (26.0)
Coronary artery disease, *n* (%)	5 (6.8)
Cerebrovascular disease, *n* (%)	2 (2.7)
Preoperative creatinine, mg/dl, mean (SD)	1.1 (0.8)
Indication for surgery:	
aortic stenosis, *n* (%)	27 (36.9)
aortic regurgitation, *n* (%)	24 (32.9)
mixed aortic disease, *n* (%)	21 (28.8)
other, *n* (%)	1 (1.4)
Bicuspid aortic valve, *n* (%)	47 (64.4)
Previous aortic valve replacement, *n* (%)	2 (2.7)
Previous aortic valve reconstruction, *n* (%)	5 (8.2)
Previous Ross procedure, *n* (%)	1 (1.4)
Previous balloon valvuloplasty, *n* (%)	1 (1.4)

## Data Availability

The presented data are available in the institutional database of Medical University of Vienna/Vienna General Hospital.
